# Indigenous medicinal plants of Pakistan used to treat skin diseases: a review

**DOI:** 10.1186/s13020-018-0210-0

**Published:** 2018-10-19

**Authors:** Alamgeer, Amber Sharif, Hira Asif, Waqas Younis, Humayun Riaz, Ishfaq Ali Bukhari, Asaad Mohamed Assiri

**Affiliations:** 1 Laboratory of Cardiovascular Research and Integrative Pharmacology, College of Pharmacy, University of Sargodha, Sargodha, Pakistan; 2 Rashid Latif College of Pharmacy, Lahore, Pakistan; 3 Department of Pharmacology, College of Medicine, King Saud University Riyadh, Riyadh, Saudi Arabia; 4 Prince Abdullah Ben Khaled Celiac Disease Research Chair, Department of Pediatrics, Faculty of Medicine, King Saud University, Riyadh, Saudi Arabia

**Keywords:** Medicinal flora, Skin problems, Ethno-medicine, Dermatological pathogens

## Abstract

**Ethno-pharmacological relevance:**

Plants are providing reliable therapy since time immemorial. Pakistan has a great diversity in medicinal flora and people use these ethno-medicines to deal with many skin problems. This review explores the fundamental knowledge on various dermatological properties of medicinal plants of Pakistan and is aimed to provide a baseline for the discovery of new plants having activities against skin issues.

**Material and method:**

A total of 244 published articles were studied using different research engines like PubMed, Google, Google-scholar and science direct.

**Results:**

Review of literature revealed ethno-pharmacological use of 545 plant species, belonging to 118 families and 355 genera, to combat various skin ailments. Out of these, ten most commonly used plant species belonging to ten different families are documented in this review. It was also found out that ehno-medicines are prepared using various parts of the plants including leaves (28.32%), whole plant and roots 13.17% and 10.97% respectively, in the form of powder (23.5%) and paste (22.75%). A total of 13 endangered plant species and ten commercially important plants were recorded.

**Conclusion:**

Medicinal plants of Pakistan have therapeutic effects against several skin problems; however most of medicinal plants are still not evaluated scientifically to support their ethno-pharmacological claim on skin. Dermatological pathogens are recommended to study. Further, the conservational programs should be established for endangered species.

## Background

History of natural products is as old as human civilizations and so is the Indigenous knowledge, which is handed down to the people from their ancestors through verbal communication; people have been living in close association with plants since time immemorial [1]. The purpose of standardizing traditional remedies is, obviously, to ensure therapeutic efficacy of medicinal plants; whereas the value of ethno-medicinal information in modern pharmacology lies in the development of new drugs. Some modern drugs have been deducted from folklore and traditional medicines [2]. The origin of the word “Ethnobotany” is accredited to US ethnobotanist “John Harshberger” who described and explained the relationship between people and plants they use in a culture in 1895 [1, 3]. In Sub-continent, Rig Veda (4500–1600 BC) compiled first record of ethno-medicine [4].

In ancient times, natural products were successfully used to treat different ailments owing to their enhanced acceptability in human society, better compatibility with the body and their natural power to treat ailment via synergistic effects and neutralizing combinations to lessen adverse effects [5]. Medicinal plants are better as compared to synthetic drugs because of minimal adverse reactions [6, 7]. Medicinal plants (trees, shrubs, grasses or vines) can be used in different forms [8] like extracts, in fresh or powdered form, seeds, fruits, vegetable mixtures, etc. [9].

According to an estimate, earth carries 265,000 species of plants but only half of these are yet investigated for their medicinal values and chemical composition. In developing countries, around 80% of the population depends upon medicinal plants for combating different diseases but this was estimated about a decade ago [10, 11] while in developed countries, 60% of the population uses these plants [6], 40–50% of the population in Germany, 42% in the USA, 48% in Australia and 49% in France depends upon plants for different health issues [12]. Importance of these medicinal plants can be judged by the fact that at least 25% of the drugs enlisted in modern Pharmacopoeia are of plant origin [5]. Also about 25% of the medical prescriptions are based on the substances or analogs of the substances of plant origin [13].

Pakistan occupies 80,943 km^2^ area and lies between 60°55′ to 75°30′ E longitude and 23°45′ to 36°50′ N latitude [14], and has altitude ranges from 0 to 8611 m with a mixed climate zones; has a large biodiversity of the medicinal plants. Pakistan is blessed with 6000 species of higher plants, of which 600–700 are used medicinally [15], out of these 6000 species, half (3000 species) are reported from Northern areas out of which 124 species have medicinal importance [16, 17], 4940 flowering plants are native to Pakistan (if cultivated flowering species are included figure turns 5738) [14]. Unfortunately only 10% of the total plant species in Pakistan have reported medicinal values [18].

In Pakistan, a large population uses folk medicines and it has become a definite part of its cultural heritage [19]. In early 1950s, most of the health concerned issues were treated using traditional indigenous experiences by more than 84% of the population of Pakistan but now this practice is limited to only remote areas of the country [6, 20, 21]. In 1958, Hocking also reported same percent (84%) of Pakistan population depending upon traditional medicinal plants for treating different ailments. In 1983, 63% of the population in Pakistan, especially in villages, was reported to use herbal medicines prescribed by the traditional prescribers [22]. Knowledge on the use of medicinal plants is enormous but if this traditional knowledge is not rapidly researched and recorded, indications are that it will be lost with succeeding generations [23].

Skin, the most diverse organ of the human body, is very important for aesthetic reasons and health issues. Skin diseases not only give unfavorable looks but also pushes the patient into psychic conditions [24]. It has been estimated that skin diseases account for 34% of all occupational disease [25]. Despite of all the developments in the medical science, it is still complicated to manage skin diseases specifically in developing countries, due to the fact that health care workers lack training in skin care and skin diseases, which have been of major concern, recently, due to their association with the Human Immunodeficiency Virus and Acquired Immunity Deficiency Syndrome (HIV/AIDS) [26]. Since human civilization, plants are used to treat major skin issues like wounds, cuts and burns [27]. In a study, it was documented that 80% of the Indian population uses ethno-medicines to deal with skin problems and 50% of the medicinal plants used against skin problems are restricted to forest [28].

This review is an attempt to summarize utmost possible information on ethno-medicines and pharmacology of the plants used in Pakistan to cure skin diseases. This study was aimed to investigate the ethno-medicinal uses of the plants of Pakistan to treat various skin conditions. We were interested in gathering the answers of the following questions in particular: Which plant species are most commonly used traditionally for skin problems? Which skin conditions are most commonly treated using ethno-medicine? Which parts of medicinally important flora of Pakistan are used against skin diseases? What are the recipes for preparing and applying the ethno-medicines? Above all, the review will identify gaps in the current knowledge that will provide a baseline for further research activities. Also, the review is aimed to highlight the area of Pakistan still need to be investigated.

## Data collection and analysis

Published papers were retrieved from the online bibliographical database latest till June 2015, search engines included: PubMed, Google, Google.scholar, IUNCredlist, druginfosys and sciencedirect. Inside the database, we used the keywords like traditional plants for skin, ethnobotany of Pakistan, ethno-medicine, traditional uses of plants, indigenous plant knowledge, plants used in ethno-pharmacology. While reviewing the literature, focus was on the plants with the potential traditional usage against various diseases of skin. In total, 244 articles on Indigenous plants of Pakistan published in English language were reviewed for this study for the period March 2015 to December 2017. Articles selected for this review contain plants that were (i) native to Pakistan with some wide distribution (ii) traditionally used in Pakistan for treating skin diseases; only those plants were selected that have ethno-pharmacological evidence for skin treatment. A master document was formulated enlisting indigenous medicinal plants of Pakistan used by the local inhabitants for the treatment of several skin ailments. Master list constituted vernacular name of the medicinal plants along with the family name, part used, mode of preparation, life form and skin conditions against which the plant is being used. All the data were summarized into 6 tables and 9 figures.

As this review is written after consulting a large number of articles, only references are provided in it due to vey diversified data.

The data collected were grouped into 26 categories on the basis of skin conditions; 28 categories based on families plant belong to; 7 categories based on mode of preparation of medicine, 30 categories based on the part used, 9 categories based on habit of plants and 6 categories based on the region the plant grows.

Plants reported in more than one region of Pakistan were enlisted only once for making the final list of medicinal plants. Conservation status was evaluated following IUCN red list categories version 3.1 and the herbal products registered in Pakistan and economical importance of the plants was estimated by consulting druginfosys.com and scientific literature respectively.

## Taxonomic problems

Several taxonomic problems were observed in the development of this manuscript. Entirely, we documented 10 plant species most frequently used by the people of Pakistan against skin diseases, there were some spelling mistakes in family names, botanical names, publication and authors, which were also verified according to “http://www.google.com”, “http://www.tropicos.org” and “http://www.plantlist.org”.

## Results and discussion

The present review revealed ethno-medicinal use of 545 plant species belonging to 118 families and 355 genera to cure various skin diseases, 10 plant species belonging to different genera and families which were most commonly used in different regions of Pakistan against several skin ailments are documented in this review (Table 1).

**Table 1:** List of plants used traditionally against skin conditions

Sr. no	Botanical name	Common name	Family	Habit	Part used	Ethnoprep	Folk claim	References	Pharmacological validation	Phyto-constituent
1	*Calotropis procera (Aiton) R. Br.*	Dasi ak, Milk weed, Akk, Spalmaka, wallow-wort, Sodom apple, Dead Sea apple	Asclepiadaceae	S	St, bk, lf, sd, rt, fr, wp	(1) Decoction of stem and leaves is taken (2) Latex is mixed with castor oil and applied (3) Paste with raw sugar is applied over the dog bitten wounds (4) Flowers are put in oil & applied on wounds (5) Crushed leaves (6) The leaves are warmed and tied over the wounds and used as poultice (7) Roots are powdered, mixed with “desi ghee” and pasted on points of leprosy	Leprosy, wound healing, abscess, ringworm, dog bitten wounds, eczema, pustules and pimples, skin eruptions, syphilis, boils, ulcer, burn, dermatitis, scabies, infection and other skin diseases	[3, 40, 58–64]	Anti-hyperbilirubinemic and wound healing activity of aqueous extract of *Calotropis procera* leaves in Wistar rats [89] *Calotropis procera* extract induces apoptosis and cell cycle arrest at G2/M phase in human skin melanoma (SK-MEL-2) cells [90] Wound-healing and potential anti-keloidal properties of the latex of *Calotropis procera* (Aiton) Asclepiadaceae in rabbits [91] Wound healing in diabetes mellitus: traditional treatment modalities [92] Healing potential of *Calotropis procera* on dermal wounds in Guinea pigs [93]	Procerursenyl acetate, proceranol, *N*-dotriacont-6-ene, glyceryl mono-oleolyl-2-phosphate, methyl myrisate, methyl behenate and glyceryl-1,2-dicapriate-3-phosphate [115]
2	Berberis lyceum Royle	Sumblu, komal, Kowdach, Berberry, Churku, Ishkeen, Ishkin (Urdu), Ishkein (Shina), Sumbal	Berberidaceae	S	Rt, st, br, lf, fr	(1) The paste of root and bark is externally applied on wounds (2) Crushed bark is soaked in water and the extract is taken in morning to treat scabies, boils and pimples (3) Decoction (4) Dried root mixed with egg and fried in cow’s ghee is used (5) Dried powdered root is spread over external wounds (6) For external wounds peel off an epidermis of root and boil the inner cortex (7) Bark powder paste mixed with mustard oil is used	Gonorrhea, wound healing, ulcers, Scabies, Boils, Pimples	[18, 65–67]	Wound healing activity of root extracts of Berberis lyceum royle in rats [94]	Berberine, ß-sitosterol, 4,4-dimethylhexadeca-3-ol, butyl-3-hydroxypropyl phthalate, 3-(4′-(6-methyl butyl) phenyl)p ropan-1-ol, 4-methyl-7-hydroxycoumarin [116]
3	*Dodonaea vescosa (L.) Jacq.*	Ghwara-sky, Sanatha, Anartirk/Hanartirk	Sapindaceae	S	Wp, Lf, sd, Bk, wd, rt, fl	(1) Paste of dried powdered leaves and water is applied (2) Fresh leaves are crushed to the extent to become sticky and then tied on the effected part of the body for wounds healing (3) Poultice (4) Leaves are grinded, mixed in water and bath is taken with this water (5) Decoction (6) Dry leaves are tied on wounds and used for softening of wound	Swelling, Germicidal, pimples, Burn and wound healing, cracked skin, rashes, itching and pustules, allergy	[40, 58, 60, 67–70]	Antifungal activity of Dodonaea viscosa Jacq extract on pathogenic fungi isolated from superficial skin infection [95] Toxicity studies on dermal application of plant extract of Dodonaea viscosa used in Ethiopian traditional medicine [96] Antifungal activity of the plant Dodonaea viscosa var. Angustifolia on Candida albicans from HIV-infected patients [97]	Sakuranetin, leucocynindns, quercetin, kaempferol, isorhamnetin, 5,7,4′-trihydroxy-3′-(3hydroxymethylbutanol) 3,6-dimethoxyflavone, 5,7-dihydroxyflavanone, 5,4′-dihydroxy-3,6,7-trimethoxy flavone, 3′-(γ,γ-dimethyalllyl)-5,7-dihydroxy-3,6,4′-trimethoxyflavone, 3,5,7,4′-Tetrahydroxyl-3,4′-dimethoxyflavone, 5,4′-dihydroxy-6,7-dimethoxyflavone, 5,7-dihydroxy-6, 4′-dimethoxyflavone, 3,5-dihydroxy-7,4′-dimethoxyflavone, 5-hydroxy-3,7,4′-trimethoxyflavone, trimethoxy flavone, 5 hydroxy-7,4′-dimethoxyflavone, 6 Hydroxy-3,6,7 trimethoxyflavone, 5,7,4′ Trihydroxy-3-methoxyflavone [117]
4	*Achyranthes aspera L.*	Apang, Puth Kanda, Prickly flower, Jishkay	Amaranthaceae	H	lf, st, sd, wp	(1) Ash of leaves and stem (2) Juice (3) Decoction (4) Paste of leaves	leprosy, Itching, Skin eruptions and irritation, abscess and boils, ulcer and other Skin diseases	[3, 36, 64, 68, 71–73]	Cancer chemopreventive activity of *Achyranthes aspera* leaves on Epstein-Barr virus activation and two-stage mouse skin carcinogenesis [98] Antibacterial activities of selected medicinal plants in traditional treatment of human wounds in Ethiopia [99] In vivo wound-healing efficacy and antioxidant activity of *Achyranthes aspera* in experimental burns [100] Evaluation of in vivo wound healing activity of methanol extract of *Achyranthes aspera* L. [101] Anti-herpes virus activities of *Achyranthes aspera*: an indian ethnomedicine, and its triterpene acid [102] Pharmacological evaluation and chemical standardization of an ayurvedic formulation for wound healing activity [103]	α-l-Rhamnopyranosyl-(1 → 4)-(β-d-glucopyranosyluronic acid)-(1 → 3)-oleanolic acid, α-l-rhamnopyranosyl-(1 → 4)-(β-d-glucopyranosyluronic acid)-(1 → 3)-oleanolic acid-28-*O*-β-d-glucopyranoside and α-l-rhamnopyranosyl-(1 → 4)-(β-d-glucopyranosyluronic acid)-(1 → 3)-oleanolic acid-28-*O*-β-d-glucopyranosyl-(1 → 4)-β-d-glucopyranoside, *n*-hexacos-14-enoic acid, strigmasta-5, 22-dien-3-β-ol, trans-13-docasenoic acid, *n*-hexacosanyl, *n*-decaniate, *n*-hexacos-17-enoic acid and n-hexacos-11-enoic acid, 36, 37-dihydroxyhenpentacontan-4-one and Triacontanol, β-d-glucopyranosyl3β-[*O*-α-l-rhamnopyranosyl-(1 → 3)-*O*-β-d-glucopyranuronosyloxy]machaerinate, β-d-glucopyranosyl3β-[*O*-β-d-galactopyranosyl-(1 → 2)-*O*-α-d-glucopyranuronosyloxy]machaerinate, β-d-glucopyranosyl-3β[*O*-α-l-rhamnopyranosyl-[1 → 3)-*O*-β-d-glucopyranuronosyloxy]oleanolate, β-d-glucopyranosyl3-β-[*O*-β-d-galactopyranosyl (1 → 2)-*O*-β-d-glucopyranuronosyloxy] oleanolate, β-d-glucopyranosyl 3β-[*O*-β-d-glucopyranuronosyloxy] Oleanolate [118]
5	*Nerium oleander L.*	Kaner, Gndeer, Ganaera, Gandeera	Apocynaceae	S	Rt, Lf, Bk, Br	(1) Oil extracted from the root and bark (2) Paste (3) Leaves with honey used as a poultice (4) Decoction	Scabies, ulcers, leprosy and scaly skin, Gangrine, maggots infesting wounds	[74–78]	Antioxidant, anti-inflammatory, anti-apoptotic, and skin regenerative properties of an Aloe vera-based extract of Nerium oleander leaves [104]	Nériine, digitoxigénine, Amorphane, 1.8-cineole, α-pinene, calarene, Limonene, βPhellandrene, Terpinene-4-ol, sabinene, Isoledene, 3-Carene, Humulene, β-Pinene and Cymen-8-ol [119]
6	*Riccinus communis L.*	Arand, Raned, Hurnoli	Euphorbiaceae	S	Wp, Sd, lf, bk, rt	(1) Grinded leaves (2) Oil obtained from the seeds (3) Paste of leaves is slightly warmed over fire and applied	Freckles, scabies, wounds and sores healing, boils, acne, leprosy, ringworm, swelling, warts removal and other skin diseases	[68]	Some Nigerian plants of de rmatologic importance [105] Antimicrobial activity of Palestinian medicinal plants against acne-inducing bacteria [106] In vitro assessment of cytotoxicity, antioxidant, and anti-inflammatory activities of *Ricinus communis* (Euphorbiaceae) Leaf Extracts [107] Natural pharmacopoeia used in traditional Toba medicine for the treatment of parasitosis and skin disorders (Central Chaco, Argentina) [108] Effect of Solanum nigrum and ricinus communis extracts on histamine and carrageenan-induced inflammation in the chicken skin [109]	Ricinine, *N*-demethylricinine, glycosides kaempferol-3-*O* kaempferol-3-*O*-β-d-glucopyranoside, quercetin Xylopyranoside, quercetin-3-*O*-β-d-glucopyranoside, kaempferol *O*-β-rutinoside and quercetin-3-*O*-β rutinoside, 1, 8-cineole, camphor and α-pinine (β-caryophyllene, ricinoleic, isoricinoleic, stearic and dihydroxystearic acids, ricinine, ester form of palmitic, stearic, arachidic, hexadecenoic, oleic, linoleic, linolenic, ricinoleic (89.4%) and dihydroxy stearic acids, ergost—en-3-ol, stigmasterol, Y-sitosterol, fucosterol; and probucol [120]
7	*Carthamus tinctorious* *L.*	Pome, Pong, Tukhmigartum, Safflower	Asteraceae	H	Fl, sd, lf	(1) Powder (2) Decoction (3) Paste (4) Juice	Chicken pox, measles and eruptive skin problems	[17, 79]	Inhibitory effects of active compounds isolated from safflower (*Carthamus tinctorius* L.) Seeds for melanogenesis [110] Kinetic study on the tyrosinase and melanin formation inhibitory activities of carthamus yellow isolated from *Carthamus tinctorius* L. [111] Inhibitory effect of hydroxysafflor yellow a on mouse skin photoaging induced by ultraviolet irradiation [112]	Enzyl-*O*-β-d-glucopyranoside, syringarenol, lirioresinol-A, 5-hydroxymethyl-2-furaldehyde, β-sitosterol, and stigmasterol [121]
8	*Solanum nigrum L.*	Mako, Black Nightshade	Solanaceae	H	Wp, Ber, lf, Ft, Lf, st	(1) The juice of the ripen berries is applied on the skin (2) Poultice (3) Crushed leaves (4) Fruits are crushed and applied externally (5) Decoction	Cosmetics, dried skin, pimples, freckles, as sun block, corrosive ulcer and suppurating Syphilitic ulcer, Pustules, ring worms, wounds healing, eczema, leukoderma	[18, 78, 80–83]	Effect of Solanum nigrum and Ricinus communis extracts on histamine and carrageenan-induced inflammation in the chicken skin [109]	(+)-pinoresinol (I), (+)-syringaresinol (II), (+)-medioresinol (III), scopoletin (IV), tetracosanoic acid (V) and beta-sitosterol [122]
9	*Albizia lebbeck (L.) Benth*	Siris, Shareen	Fabaceae	T	Lf, sd, bk, fl, st, wd, tr	(1) Dried leaves are smoked (2) Paste of flower (3) Extract	Wound healing, leucoderma, itching, Inflammations, boils, eruption	[78, 84]	Wound-healing potential of the root extract of Albizzia lebbeck [113]	Budmunchiamines L1–L3, Quercetin, kaempferol, 3-*O*-α-rhamnopyranosyl (1 → 6)-β-glucopyranosyl(1 → 6)-β-galactopyranosides and Albiziasaponins A, B and C [123]
10	*Plantago lanceolata L.*	Bar-e-Thang, Boieko-ligini, Isphaghol, Ghwa jabai	Plantaginaceae	H	Lf, ft, sd, rt	(1) Fresh leaves are mashed and put on wounds	Wound and burn healing, Skin sores, inflamed surfaces, bruises	[3, 4, 66, 85–88]	*Plantago* *lanceolata* L. Water extract induces transition of fibroblasts into myofibroblasts and increases tensile strength of healing skin wounds [114]	catalpol, aucubin, and acteoside [124]

H, herb; S, stem; T, tree; Bk, bark; Lf, leaf; sd, seed; rt, root; Wp, whole plant; ber, berries; fl, flower; br, branch

### Distribution of plants in different regions of Pakistan

**Fig. 1:**
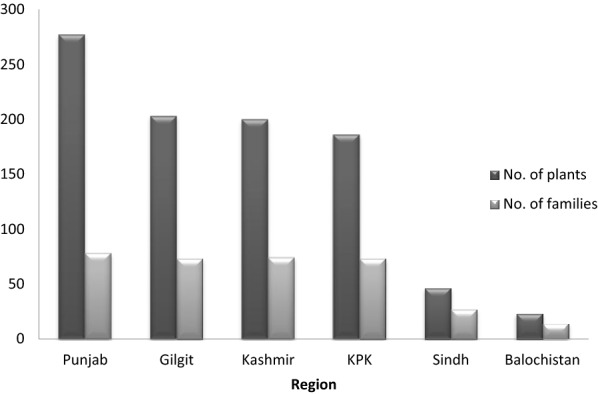
Distribution of medicinal plants in different regions of Pakistan

Pakistan holds rich diversity of medicinal plants used against various ailments. The present review reported 545 plants from different regions of Pakistan being ethno-medicinally used for treating various skin problems. Majority of 278 plants were reported from Punjab belonging to 78 families followed by 204 plants from Gilgit from 73 families, 201 from Kashmir belonging to 74 families, 187 from KPK from 73 families, 47 from Sindh from 27 families and 25 from Balochistan belonging to 14 families. Many regions are still un-investigated; a list of investigated as well as un-investigated areas of Pakistan is given in Table 2. Many of the plants and hence families were used in more than one region; those plants and their families were counted just one time when enlisting for overall plants of Pakistan. The distribution of plants and their families in different regions of Pakistan, according to their use in skin conditions is shown in Fig. 1.

**Table 2:** Investigated and under-investigated districts for ethno-botanical studies

Province	Investigated districts for ethno-botanical studies	Under-investigated districts for ethno-botanical studies
*Punjab*	Attock, Bahawalnaga, Bahawalpur, Bhakkar, Chakwal, Dera Ghazi Khan, Faisalabad, Gujranwala, Gujrat, Jhang, Jhelum, Kasur, Khushab, Mianwali, Multan, Muzaffargarh, Narowal, Nankana Sahib, Pakpattan, Rajanpur, Rawalpindi, Sahiwal, Sargodha, Sialkot, Toba Tek Singh, Vehari	Chiniot, Hfizabad, Khanewal, Okara, Rahim Yar Khan, Sheikhupura
*Khyber Pakhtunkhwa*	Abbottabad, Bannu, Battagram, Buner, Chitral, Dera Ismail Khan, Haripur, Karak, Kohat, Upper Kohistan, Lakki Marwat, Lower Dir, Malakand, Mansehra, Peshawar, Swat, Upper Dir, Lower Kohstan	Charsadda, Hangu, Mardan, Nowshera, Shangla, Swabi, Tank, Tor Ghar
*Sindh*	Ghotki, Jamshoro, Karachi, Kairpur, Sanghar, Sukkur, Tharparkar, Thatta, Karachi West	Badin, Dadu, Hyderabad, Jacobabad, Kashmore, Larkana, Matiari, Mirpurkhas, Naushahro Firoze, Shaheed Benazirabad. Kambar, Shahadkot, Shikarpur, Tando Allahyar, Tando Muhammad Khan, Umerkot, Sujawal, Karachi Central, Karachi East, Karachi South, Korangi, Malir
*Sindh*	Ghanche, Skardu, Astore, Diamer, Ghizer, Gilgit, Hunzanagar	Kharmang
*Gilgit* *Baltistan*	Muzaffarabad, Hattian, Neelum, Mirpur, Bhimber, Kotli, Poonch, Bagh, Haveli, Sudhnati

### Medicinally important families and genus against skin ailments

A total number of 118 families were studied and out of them 10 families were documented in this review. Among these families, Asteraceae (8.99%) is the most commonly used family followed by Fabaceae (4.58%), Poaceae (3.73%), Laminaceae and Ranunculaceae (3.39% each), Rosaceae (3.22%), Euphorbiaceae and Solanaceae (3.05%), Polygonaceae (2.88%), Boraginaeae (2.71%), Papilionaceae and Amaranthaceae (2.37% each), Apocynaceae (2.20%), Cucurbitaceae and Malvaceae (1.86.5% each), Chenopodiaceae, Salicaceae and Liliaceae (1.69% each), Apiaceae and Brassicaceae (1.52% each), Moraceae, Scrophulariaceae and Zygophyllaceae (1.35% each) Capparidaceae, Convolvulaceae and Rutaceae (1.18% each), Cyperaceae, Rhamnaceae, Saxifragaceae and Tamaricaceae (1.01% each) and some other 87 families (30.39%).

**Fig. 2:**
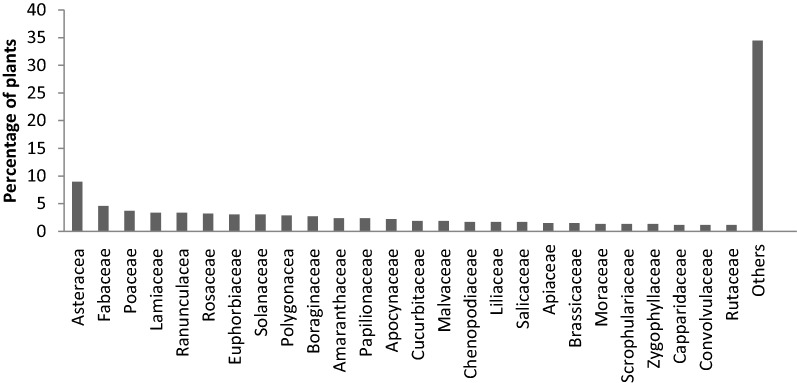
Families of plants active against skin ailment

The results, in terms of percentage, of commonly used families are represented in Fig. 2.

Asteraceae holds the top position among the families used in ethno-medicines which indicates the presence of effective bioactive ingredients in the members of this family [29]. Lamiaceae and Asteraceae are the most frequently used families in ethno-medicine [30]. It was reported that Asteraceae family is the most diverse family found in all the habitat and regions except in Antarctica and it was not a new finding about Asteraceae family holding highest position among the traditionally important families, it was further concluded that this prevalence is due to high quantities of active secondary metabolites present in this family and also because this family includes a large number of species [31].

Euphorbia is the genera having highest number of species (11 ssp.) used to fight skin problems followed by Artemisia with 9 ssp., Ficus and Salix (8 ssp. each), Solanum (7 ssp.), Impatiens, Polygonum, Rumax, Saussurea, Ziziphus (6 ssp. each), Clematis, Datura, Tamarix, Vernonia (5 ssp. each), 15 genera with 4 ssp., 23 genera with 3 ssp., 72 genera with 2 ssp. and 224 genera with 1 ssp. used to combat different skin ailments.

**Fig. 3:**
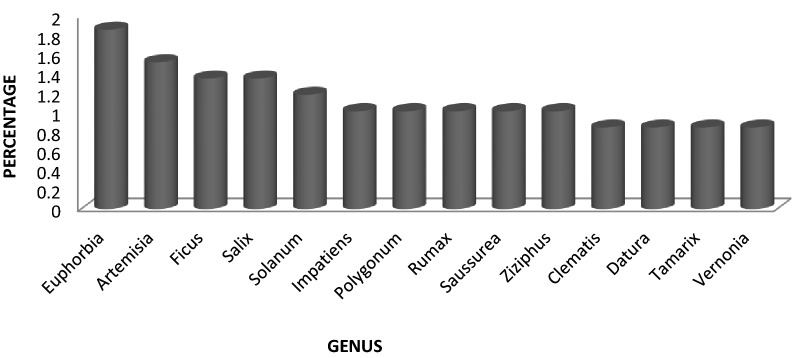
Common genera used for skin diseases

Medicinally important genera along with the number of species used effectively against skin ailments in terms of percentage are graphically represented in Fig. 3.

Euphorbia taking the top place in the genera used most frequently in ethno-medicine due to the reason that it is the largest genus of family Euphorbiaceae with 1600 reported species [32].

### Common skin diseases in Pakistan

In this review, it was found out that most of the plant species were used to treat more than 1 disease/condition of skin. Total 26 skin conditions were reviewed which were treated using ethno-medicine. Most common of them was wound healing (17.064%) followed by boil healing (8.72%), postules or pimples (5.83%), eczema (5.01%), ulcer (4.579%), burn (4.51%), Ringworm (3.889%) and scabies (3.889%).

**Fig. 4:**
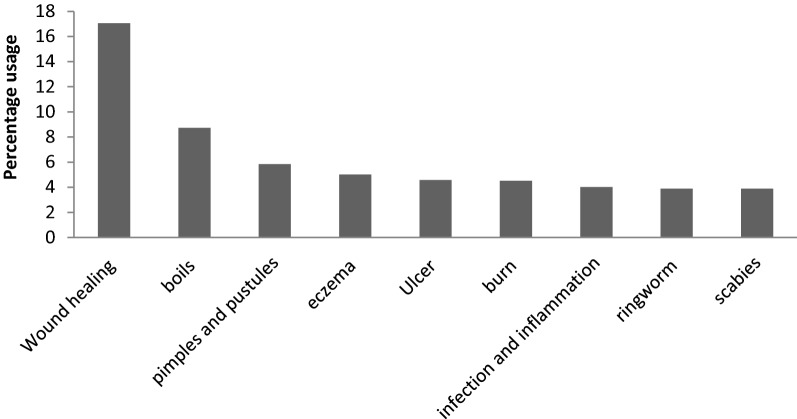
Common skin diseases treated traditionally

**Fig. 5:**
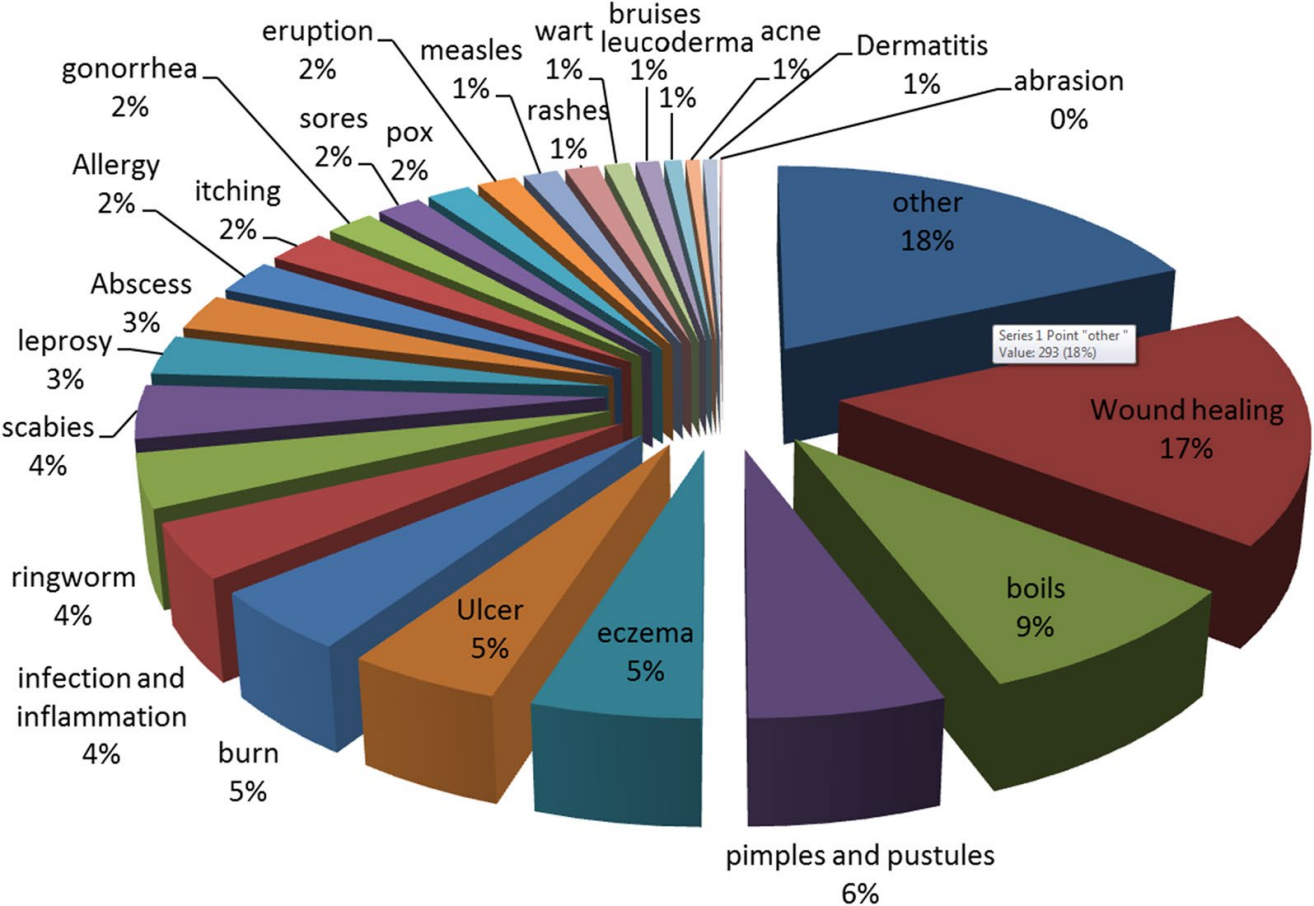
All the skin diseases treated traditionally in Pakistan

These most commonly treated skin conditions mentioned above are represented graphically in Fig. 4 and total of all the skin conditions treated traditionally are represented in Fig. 5.

Burn, which accounts 4.51% of the skin conditions treated traditionally in Pakistan is a serious issue globally, De Wet et al. reported increased mortality rate due to burn which has contributed to a serious health concerned issue globally. In South Africa, burn is one of the major reasons of deaths among 5–29 years individuals. Ethno-medicines have great potential to cure different kind of skin diseases [25, 137].

Some plants cure more than one skin disease, *Curcuma longa* and *Melia azaderach* are the natural resources for curing multiple skin diseases [33, 138].

Tabassum and Hamdani concluded that skin conditions can be of thousand types but are categorized into nine categories based on common diseases; rashes, viral infections, bacterial infections, fungal infections, parasitic infections, pigmentation disorder, tumor and cancer, trauma and other conditions (Wrinkles, spider veins and varicose veins) [28]. This review revealed that all these skin conditions are treated traditionally using etho-medicines.

Mabona and Vuuren reported wound healing as a most frequently treated skin condition using ethno-medicine, followed by sores or ulcers. Among the medicinal plants used to combat wounds, Erythrina genus is used most frequently in South Africa with 120 species having their space in the traditional usage against skin conditions particularly in disinfection of wounds. Traditional plants are also used to combat associated condition of wounds including inflammation, urticaria, skin allergies, acne, eczema and psoriasis. Among these associated conditions, eczema is most commonly diagnosed in South Africa and thus most frequently treated with ethno-medicines [34].

### Habit

**Fig. 6:**
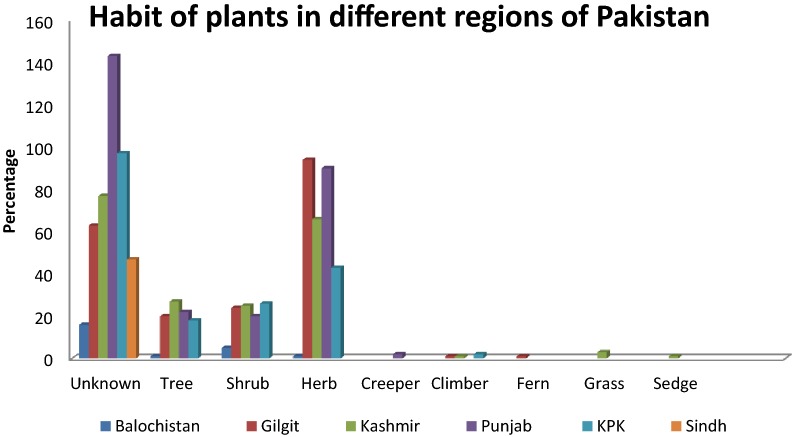
Habit of plants in different regions of Pakistan

**Fig. 7:**
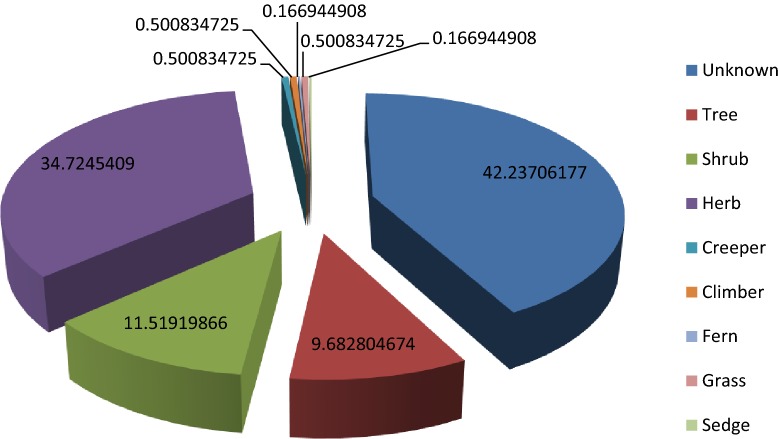
Habit of plants in Pakistan

Growing habits of 42.23% plants were not available in the previous articles. Common life forms used by traditional healers for the preparation of ethno-medicines include Trees 9.68%, Shrub 11.52%, Herb 34.72%, Creepers and Climbers 0.5% each, Grass 0.5% (only 3 plants were a grass), Sedges 0.1% (only 1 plant was from sedges) as shown in Figs. 6 and 7.

Trees and shrubs are used to prepare majority of herbal recipes as they are accessible round the year, followed by utilization of herbs which might be related to their easy collection methods, higher abundance and efficacy in curing ailments as compared to other life forms [30]. Khan et al. reported shrubs and trees as most commonly used life forms in ethno-medicines due to the their availability around the year with the minimum seasonal variation [35]. Ahmad et al. stated that herb is used in ethno-medicines due to their abundance and easy availabilty in most of the aresa of the world [36].

### Part used

**Fig. 8:**
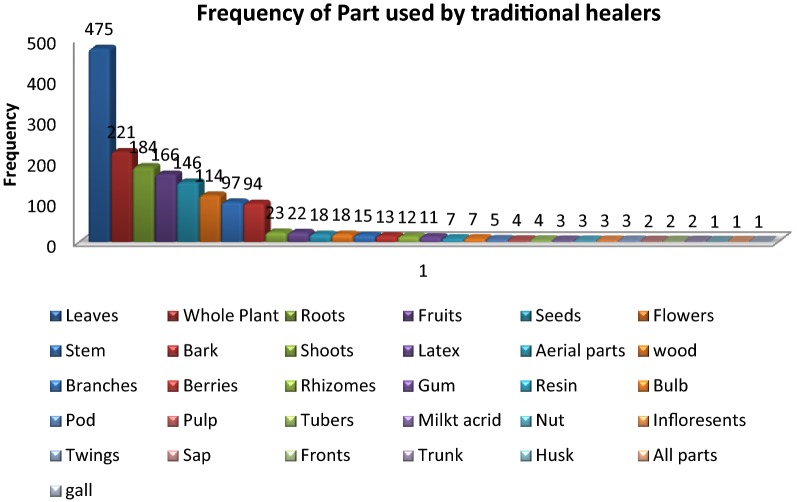
Part used by traditional healers to prevent different skin ailments

Ethno-medicines mostly consisted of plant parts such as leaves, roots, fruits, seeds, flowers, stem, bark and some other parts of the plants listed below. Traditional knowledge on the effectiveness of different plant parts could have been established through trial and error basis along with observations such as taste, smell and texture. This study revealed that leaves (28.32%) were the most common part of indigenous plants used in different preparations of ethno-medicines for the treatment of skin diseases followed by whole plant (13.17%), roots (10.97%), fruits (9.89%), flowers (6.79%), stem (5.78%), bark (5.60%), Shoos and latex (1.37% each), aerial parts and wood (1.07% each), branches (0.89%), Berries (0.77%), rhizomes (0.71%), gum (0.65%), resins and bulb (0.41% each), pod (0.29%), pulp and tubers (0.23% each), milky acrid, nut, inflorescent, twigs (0.17% each), sap, fronts, trunk (0.11% each), husk and gall (0.05% each) (Fig. 8).

Leaves were the most commonly used plant part in ethno-medicine, this is due to the ease of processing them into a digestible paste and have less conservational issues than the collection of roots, bark, stem or the whole plant and also because leaves does not affect the life cycle of the plants [29], further the life form of the plant is not effected by the collection of leaves [37] and also due to the reason that leaves contain photosynthate which might have some medicinal value [38]. Ayyanar and Ignacimuthu reported leaves as most frequently used plant part (accounting 50%) to prepare ethno-medicines in Kani Tribes, India [39].

Whole plant and roots are other frequently used plant parts probably due to bioactive components enriched in these parts. However, their excessive use is detrimental for their survival since whole plant has to be uprooted. Not only roots, even the use of more than one plant part for medicinal purpose has put these plants to extinction risk owing to damage inflicted on the plants [40].

### Mode of preparation

Herbal preparation is made by using different plant parts like whole plant, leaves, roots, stem, fruits, flowers, barks, berries and seeds.

Different modes of preparation are used for different plants by using various parts by the native people; this study showed 23.5% ethno-preparations were used in powdered form, 22.7% paste, 16.37% decoction, 12.62% juice, 8.75% poultice, and 8% each extract and infusion and 16.25% were not mentioned in literature.

**Fig. 9:**
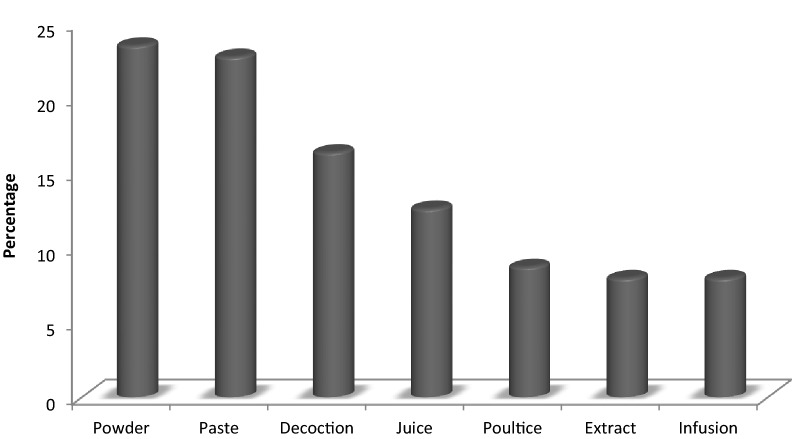
Mode of preparation of plant

Different modes of preparation of the plants are shown in Fig. 9.

The missing information regarding mode of preparation of the ethno-medicine was also reported by Farnsworth who indicated the published studies include raw list of plants and only a small number of manuscripts reports the mode of preparation while the rest indicates the part used in treating a particular ailment [41].

Literature revealed that plants can be used in different ethno formulations depending upon the sort of skin disease to be treated and the type and part of plant selected to treat the disease. The ethno-formulation used for skin condition may include powder, paste, plant juice, ointment, poultice, extract, decoction or infusion [42, 43].

Decoction and infusion are frequently used in ethno-medicine due to the ease of preparation and because water is used as a solvent in these formulations, which is easily available [34]. Felhaber reported that ethno-medicines can be given orally or applied topically. Though both the routes can be used but topical application is common as well as effective as it ensures direct contact of active constituents of plant with the site of action (skin) and also because it gives a quick relief [44].

### Combined ethno-preparation

Medicinal plants are used in combination with other medicinal plants, with vegetable oils (mustard oil, apricot oil etc.) and/or with the nutrients (milk, ghee etc.). People of Pakistan still rely on synergistic interactions of plants and supportive components like oil and milk to treat skin diseases.

These supplement ingredients may be used to enhance the effect of the herbal preparations or are simply used to make the preparations palatable. And on the other hand they improve the healing conditions; however, the exact role of these materials in curing the diseases is not clearly known [29].

A number of plant combinations used to treat skin conditions have been reported. The synergistic interaction of medicinal plants have been used since antiquity. Mabona and Van Vuuren reported that medicinal plants are used alone as well as in combination with other plants without any adequate validation of the combination. Plant species like *Acorus calamus, Cyathula natalensis, Cyanella lutea, Hypoxis latifolia, Momordica foetida, Pittosporum viridiflorum* and *Vernonia natalensis* have effects on skin when used in combination with other plants but if used alone, they do not have any dermatological effects; they may have potential to treat the additional symptoms associated with skin conditions like fever in skin infection [34].

Plants used in combination with other plants are given in Table 3, plants used in combination with different oils in Table 4 and plants used in combination with nutrients are given in Table 5.

**Table 3:** Combination of ethno plants used for skin conditions

Sr. no.	Combination	Mode of preperation	Folk claim	References
1	*Cynodon dactylon (L.) *+* Curcuma longa L.*	Grind along with *Curcuma longa* and rice	To stop bleeding from wounds, scabies, fungal infections, leprosy, wound healing, pimples, eczema, anti-septic, itching, small pox	[40]
2	*Melia azedarach L. *+* Phyllanthus emblica* L. +* Terminalia chebula Retz.*	Leaves are ground in water along with black pepper. Tablet are prepared from paste of dry friut of Melia azadarach, Phyllanthus emblica, and Terminalia chebula	Skin infection, wound healing and leprosy, scabies, pustules, pimples, boils and other skin diseases, allergy and itching, eczema and measles, boil, allergy, leucoderma, Cutaneous infections, Germicidal	[67]
3	*Aloe vera (L.) *+* Citrus limon (L.) Burm. f.*	Pulp of aloe along with 2–3 drops of lemon and rose extract is also used	Wound healing, chronic, dermatitis and radiation burn treatment, cuts, burns, eczema, sunburn, dermatitis, acne, freckles, pimples and boils, ulcer	[125]
4	*Aloe barbadensis Mill. *+* Curcuma longa L. Aloe barbadensis Mill. *+* Cichorium intybus L.*	Pulp of the leaf of Aloe is added with powdered *Curcuma longa* for dermal use on wounds. Pulp of the leaf of Aloe and chicory (*Cichorium intybus*) are soaked in water at night	Skin beauty, skin disease, wound healing, burn, eruption, boils, itchy skin	[40]
5	*Cyperus rotundus L. *+* Curcuma longa L.*	Roots of *Cyperus rotundus L*. along with turmeric and curd are made into a paste	leprosy, Skin diseases, pruritus, eczema, wound healing, pimples, acne	[126]
6	*Xanthium stromarium Linn. *+* Calotropis procera* (Aiton) R. Br	Fresh leaves of *Xanthium stromarium* Linn. are crushed with the leaves of *Calotropis procera*	leprosy, and purities, wound healing, Smallpox, eczema, Ulcers, boils	[75]
7	*Canabis sativa Linn *+* Cassia fistula Schimp.ex.Oliv*	The seed of *Cassia fistula* and one gram leaves of *Cannabis sativa* are taken along a cup of milk	Leprosy, Wound healing, Measles, Dressing of wounds and sores, Scabies	[68]
8	*Clematis orientalis L. *+* Pine*Wolf	The leaf paste used with resin of Pine on wounds to cure promptly	Antiseptic, pimples, antiseptic, burns, Eczema, Wound healing, Syphilis	[127]
9	*Chenopodium album L. *+* Solanum surattense Burm. f.*	Decoction of *Chenopodium albumis* is prepared with *Solanum suttattense*	Ulcer, Measles and other skin diseases	[128]
10	*Fragaria nubicola (Hook. f.) nubicola (Hook. f.) *+* Berberis lyceum Royle*	Powder of leaves and roots is mixed *Berberis lycium*	Skin infections	[129]

**Table 4:** Plants used in combination with oils

Botanical name	Common name	Family	Part used	Ethno preparation	Folk claim	References
*Acacia Arabica* (Lam.) Willd.	Kekar	Fabaceae	F, L, S, Bk	mixed with oil And applied	Wound healing, eczema and other skin diseases	[40]
*Aconitum heterophyllum Wall. ex Royle*	Zhadwar	Ranunculaceae	R, Fl	The dried pulverized roots are mixed with butter oil	Abscess, boils and other skin diseases	[130]
*adhatoda zeylanica Medik*	Bhaikar, Barg-e-baansa and Arrusa	Acanthaceae	WP	Ash of leaves is mixed with mustard oil and externally	Pustules and pimples	[69]
*Allium cepa L.*	Ghashoo, Tukhm peyaz, onion	Liliaceae	S, L, bk, bu	One or two scales are warmed in mustard oil and crushed. This paste is applied twice a day for 2–3 days	Wound healing, Eruption, boils, Chicken pox, Pimples, Skin infections, gonorrhea and other skin diseases	[67]
*Cleome viscosa Linn.*	Kinni Buti	Capparidaceae	L, Sd	Leaves are coated with sesame oil and warmed over fire, which are applied over pain and inflammation of the boils	Boils, ringworm	[126]
*Calotropis procera (Willd.) R. Br.*	Dasi ak, Milk weed, Akk, Spalmaka, wallow-wort, Sodom apple, Dead Sea apple	Asclepiadaceae	S, Bk, L, Sd, R, Ft, WP	Latex is mixed with castor oil and applied on skin. Flowers are put in oil applied to wounds to cure them. Roots are powdered, mixed with “desi ghee” and pasted on points of leprosy	Leprosy, wound healing, abscess, ringworm, eczema, pustules and pimples, eruptions, syphilis, boils, ulcer, burn, dermatitis, scabies, skin infection	[8]
*Lawsonia alba Lam.*	Mehhndi	Lythraceae	L, Ft	Leaves are mixed with the mustard oil and made into paste	Leukoderma, athlete foot	[62]
*Polygonum tataricum L.*	Bro Kho-Bro	Polygonaceae	L, Sd	The flour is mixed with apricot oil in boiling water; paste is formed and applied to skin overnight.	Skin disorders	[131]
*Potentilla nepalensis Hook.*	Panzpatar, Rattenjot	Rosaceae	L, R	The leaves are boiled in milk and applied/powder (ashes) is being applied with oil	Boil healing, burns and bruises	[22]
*Sporobolus ioclados (Trin.)*	Ganri	Poaceae	Sd, L	Mixed with oil and applied	Scabies	[129]

Bk, bark; Bu, bulb; F, fruit; Fl, flowers; Ft, fruit; In, inflorescence; L, leaf; Pd, pod; R, roots; S, stem; WP, whole plant

**Table 5:** Plants used in combination with nutrients

Botanical name	Common name	Family	Part used	Ethno-preperation	Folk claim	References
*Ajuga bracteosa Wall. ex Benth*	Khawaja booti,, Kauri Booti	Lamiaceae	L, R, WP	Macerate with yogurt is applied	Skin infections, Wound healing, pimples, measles, Boils	[63]
*Zizipnus manurithiana Lam.*	Gundar	Rhamnaceae	L	Leaves are poused into a paste mixed with gurh and then applied as a poultice	Abscess	[132]
*Bergenia ciliate A. Br. ex Engl.*	Chata pana (Zakhm-e-hayat)	Saxifragaceae	R, Fl, L, Bk, Sd, Rh	The powder of roots is mixed with Desi ghee	Wound healing, Sun burn, washing ulcers, infection, pimples and other skin diseases	[65]
*Boerhaavia diffusa L.*	Ensut	Nyctaginaceae	R	Roots are crushed, boiled in milk and poultice is made	Ulcer	[133]
*Citrus medica Linn.*	Khatti, Naranj	Rutaceae	L, Sd, Lt	Fruit extract is mixed with honey and fresh milk to make its paste	Itching, inflammation, Pimples	[6]
*Convolvolus arvensis Linn*	Heran Kari, Heran-paddi	Convolvulaceae	P, Sh, L, F, R, S, Fl	Plant is ground along with black pepper and eaten	Wound healing, boils, scabies, ringworm	[134]
*Cynoglossum denticulatum DC*	Pitrus	Boraginaceae	R	Root is powdered and mixed with milk	Pustules and pimples	[69]
*Ficus variegata Wall. Ex Roxb*	Phagwara	Moraceae	WP	Paste prepared from fresh milky juice of plant mixed milk	Boils, infected skin	[67]
*Grewia tenax (Forssk.) Fiori*	White spurry, Kaankeh, Wingo	Tiliaceae	L	The ash of the leaves is mixed with butter to make poultice	Abscess and wound healing, boils	[135]
*Jurinea macrocephala Benth. ex Hook.f.*	Gogol Doop	Asteraceae	R	Decoction of roots is mixed with butter	Burn	[130]
*Mallotus philippensis (Lam.) Müll. Arg.*	Kambeela	Euphorbiaceae	F	Red powder of fruit is mixed with butter	Ezcema	[136]
*Otostegia limbata* (Benth.) Boiss	Kori booi, Spin Azghay, Chiti, Chittakanda, Ghawareja	Lamiaceae	L	Dried leaves are ground and mixed in butter to form a paste. Leaves are dried, ground and powder is mixed with honey. A table spoon is taken once a day	Wound healing and bruises	[22]
*Pinus roxburghii Sarg*	Chir, Nakhtar	Pinaceae	AP, Sh, G, Bk, Rs	Young shoot is fried on pan and milk is added	Wounds, sores, burns, boils and ulcers, measles	[69]
*Pinus wallichiana A. B. Jacks*	Cheenh, Biar, Kail	Pinaceae	Rs, W	Resin admixture with honey	Wound healing, antiseptic, Gonorrhea, abscess, for burning sensation, ulcer	[127]
*Rumex nepalensis Spreng*	Rawas, Hoola	Polygonaceae	L, S, R	Dried leaf powder is mixed with butter (ghee)	Wound healing and anti-allergy, boil	[127]
*Stellaria media (Linn.)*	Khashkhash boti, Losdhi, Salooni booti	Caryophyllaceae	WP, Sd, L	Seed powder with milk is given to children to cure skin infection and allergy	Wound healing, infections, allergy, itching-skin condition, eczema, ulcers, boils, abscesses, rashes, burns and irritations	[128]
*Viola serpens Wall*	Banafsha	Violaceae	WP, Fl	The whole plant is taken and boiled in milk till it become gelatinous. Bandage is made from it and used as poultice	Wound healing, eczema	[129]

Ac, milky acrid; AP, aerial parts; Bk, bark; Bu, bulb; F, fruit; Fl, flowers; Ft, fruit; G, gum; In, inflorescence; L, leaf; Lt, latex; P, Petals; Pd, pod; R, roots; Rh, rhizomes; Rs, resin; S, stem; Sh, shoots; W, wood; WP, whole plant

### Endangered species

Endangered medicinal plants of Pakistan that have got their role in treating skin conditions are discussed in Table 6.

**Table 6:** Endangered species of Pakistan

Sr. no	Botanical name	Family	Category	Major threat	Conservation action	References
1	*Aconitum chasmanthum Stapf ex Holmes*	Ranunculaceae	CR	I. Over harvesting and loss of habitat due to construction of high altitude roads and avalanch II. Regeneration of the species is hampered due to unsustainable collection of tubers and roots	I. Intensive studies on the population trend, reproductive biology and propagation techniques should be carried out to support conservation action programs II. Habitat managment and sustainable collection practice III. Active in situ conservation may be undertaken in protected areas IV.Surveying and monitoring is needed across the known range of occurrence to ascertain the status of wild subpopulations	http://www.iucnredlist.org/details/50126558/0 (assessed on 25.12.2016)
2	*Aconitum heterophyllum Wall. ex Royle*	Ranunculaceae	EN	I. Loss of habitat due to road construction and unsustainable collection from wild II. Large scale collection III. This species is under severe threat due to illegal collection and marketing	I. Intensive studies on the population trend, reproductive biology and propagation techniques should be carried out to support conservation action programs II. Habitat managment and sustainable collection practice III. Active in situ conservation may be undertaken in protected areas IV. Surveying and monitoring is needed across the known range of occurrence to ascertain the status of wild subpopulations	http://www.iucnredlist.org/details/50126560/0 (assessed on 25.12.2016)
3	*Aconitum violaceum Jacquem. ex Stapf*	Ranunculaceae	VU	I. Loss of habitat due to agriculture and unsustainable II. Collection from wild	I. Habitat loss and over exploitation II. It needs immediate attention in habitat management and sustainable collection practices III. Active in situ conservation should be undertaken in protected areas IV. Surveying and monitoring is also needed throughout the known historic range of the taxon to ascertain the status of all recorded subpopulations V. Intensive studies on population trend, reproductive biology and propagation techniques need to be carried out to support conservation action programs	http://www.iucnredlist.org/details/50126560 (assessed on 25.12.2016)
4	*Betula utilis D. Don*	Batulaceae	LC	I. Over exploitation as it is a high value medicinal plant II. In the Mankial Valley Hindukush Range, Pakistan, 85% of the population has decreased	Harvesting must be sustainable to ensure the survival of this species	http://www.iucnredlist.org/details/194535/0 (assessed on 25.12.2016)
5	*Cedrus deodara (Roxb. ex D. Don) G. Don*	Pinaceae	LC	I. Intensive logging (legal and illegal) II. Deforestation and conversion of forests for agriculture may also pose local threats in some parts of Pakistan and India	Known from several protected areas across its range	http://www.iucnredlist.org/details/42304/0 (assessed on 25.12.2016)
6	*Commiphora wightii (Arn.) Bhandari*	Burseraceae	CR	I. Unsustainable collection of multiple parts, high volume trade and loss of habitat II. Grazing and browsing by sheep and goats III. Collection of branches as fuel wood during the rainy season, scarcity or festive times IV. This species demonstrates one of the most generic problems of conservation V. Overexploitation, a narrow extent of occurrence, small area of occupancy, severe fragmentation of populations, very low regeneration and invasion of alien species mean that *C. wightii* is facing a high extinction risk	I. Biotic pressure should be regulated II. Standard and better gum extraction technique could minimize the mortality rate of the species (Dixit and Rao 2000) III. *Ex situ* conservation and multiplication through micro and macro propagation technique IV. Some attention and efforts have been brought into the system by identifying and documenting more than 100 forest areas (MPCAs)	http://www.iucnredlist.org/details/31231/0 (assessed on 25.12.2016)
7	*Ephedra intermedia* *Schrenk ex C.A. Mey.*	Ephedraceae	LC	Over harvesting should be investigated	Monitoring of wild harvesting is recommended to better understand how this is affecting population size	http://www.iucnredlist.org/details/201664/0 (assessed on 25.12.2016)
8	*Gentiana kurroo Royle*	Gentianaceae	CR	I. Loss of habitat and unregulated harvesting. Due to road construction and agricultural invasion II. Over grazing and human settlements III. Climate change, in terms of temperature and rainfall, has severe impacts on the population and habitat	I. It needs immediate attention in terms of trade regulation, habitat management and sustainable collection practice II. Ex situ conservation and cultivation may help to reduce the pressure on wild population	http://www.iucnredlist.org/details/50126594/0 (assessed on 25.12.2016)
9	*Juniperus excels M. Bieb.*	Cupressaceae	LC	No specific range wide threats have been identified for this species; over exploitation and habitat degradation and conversion may be localised problems	This species is known from several protected areas throughout its range	http://www.iucnredlist.org/details/42232/0 (assessed on 25.12.2016)
10	*Juniperus squamata Lamb.*	Cupressaceae	LC	Overgrazing	This species is recorded from many protected areas	http://www.iucnredlist.org/details/42254/0 (assessed on 25.12.2016)
11	*Pinus gerardiana Wall. Ex D. Don*	Pinaceae	NT	I. Conversion for pine forests for agricultural use, increasing the degree of fragmentation, and overgrazing that prevents natural regeneration II. Over harvesting of seed cones contributes to poor regeneration III. Over exploitation for firewood	I. In Afghanistan, plantations have been established to supply the seeds II. In other parts of its range *P. gerardiana* forests are included within protected areas III. A combination of reafforestation programmes coupled with sustainable use strategies are needed before this species declines sufficiently to become eligible for a threatened category	http://www.iucnredlist.org/details/34189/0 (assessed on 25.12.2016)
12	*Pinus roxburghii Sarg.*	Pinaceae	LC	While forest destruction and logging have reduced the area of occupancy (AOO) of *P. roxburghii*, it is still covering extensive areas (an estimated 0.87 million ha in India alone) and is therefore not considered to be threatened with extinction. Improved methods of resin tapping have decreased the risk of trees dying prematurely	This species occurs in some protected areas	http://www.iucnredlist.org/details/42412/0 (assessed on 25.12.2016)
13	*Pinus wallichiana A.B. Jacks.*	Pinaceae	LC	Potentially, over-exploitation could negatively impact the population, but the species is too common and wide-spread for this to have serious consequences other than locally	This species occurs in several protected areas	http://www.iucnredlist.org/details/42427/0 (assessed on 25.12.2016)

CR, critically endangered; EN, endangered; LC, least concern; NT, near threat; VU, vulnerable

Using the part like roots, rhizomes or bulbs could be a severe threat for reproduction of medicinal plants of the area. The plants collected by using these methods, especially those propagated through rhizome, bulb or corm, need sustainable utilization and conservation strategies. Un-sustainability of harvesting of herbaceous roots is well recognized by conservationists and termed such medicinal plants as highly threatened [40]. Uprooting a plants is the most detrimental method of plant collection, if the roots are not removed completely it can also result in destruction by decreasing water upset and increasing susceptibility of fungal infection. Commercialization is the major cause of extinction of medicinal plants in South Africa which demands over harvesting and thus has taken natural medicinal resources to near extinction [45].

Shanwari suggested to establish protocols to interpret the pattern of plant growth and to accelerate the knowledge about propagation of medicinal plants and avoiding harvesting of wild species in order to save endangered species [15].

*Aconitum chasmanthus*, *A. heterophyllum*and*, A. violaceum*, which are native to Pakistan, India and Nepal [46–48], are harvested for their tubers which constitute Ayurveda drug and for this reason whole plants are uprooted. In 2003 in a CAMP workshop organized at India (Shimla), experts agreed that 80% of the wild population of *Aconitum chasmanthus* had declined and therefore it is assessed as “critically endangered species”, 70% of that of *A. heterophyllum* had declined and it is assessed as “endangered” while 40% decline of the population of *A. violaceum* has made it a “vulnerable” specie to become endangered. This decline is over a decade but the situation has not improved and therefore the status id still valid [49]. It was observed in a survey in India that collection amount of *A. heterophyllum* has dropped down from 200 g per person per day to 70–100 g per person per day in 5 years [50]. Moreover, illegal collection of this specie has let it threatened [51].

Reddy et al. reported that oleo-gum resin tapped from the stems of *Commiphora wightii* constitutes the well-known Ayurvedic drug “Guggul” which is consumed in high volumes by the herbal industries of Sub-continent. Field observations over the last several decades have confirmed a severe decline in its wild population, as the shrubs tapped for oleo-gum resin die within 2–3 years. Over the past 84 years (three generation lengths) there has been a decline of more than 80% in the wild population as a result of habitat loss and degradation, coupled with unregulated harvesting and tapping of oleo-gum resin. This species is therefore assessed as Critically Endangered [52]. Soni identified a number of relevant activities within the study area under the theme ‘Guggal Bachao Abhiyan’ (Save Guggal Movement). These were conducted through the close co-operation of the village level communities, who depend on local biodiversity for their livelihoods in the Aravali Hills of Rajasthan [53].

*Gentiana kurroo* is mainly collected from the wild and there is no information regarding its cultivation. Hence, the species is under severe threat of extinction. This assessment was primarily based on the very limited reported presence of the species in wild and the high demand and prices for the dried roots of this plant. The wild population in IndoPak region is inferred to have declined by 80% in a 10 years time period. The recent CAMP assessment also agrees with the trend of population decline of more than 80% in India. Therefore, the species is assessed as Critically Endangered [54, 55].

### Commercially available important plants

Plants that are available commercially in Pakistan in different formulations are enlisted in Table 7.

**Table 7:** List of some commercially available medicinal plants used in Pakistan

Sr. no	Botanical name	Brand name	Manufacturer	Ingredients	Dosage form	Dosage
1	*Calotropis procera (Willd.) R. Br.*	PACHNOL	Hamdard laboratories Waqf Pakistan	Ammonium chloride68.070000 mg/Tab *Calotropis procera* (*Ait.) R. Br*.11.345000 mg/Tab *Ferula assafoetida* Linn.5.627000 mg/Tab Lake salt68.070000 mg/Tab *Myrtus caryophyllus Spreng.* 84.952000 mg/Tab *Piper nigrum Linn*.34.035000 mg/Tab Potassium Carbonate11.345000 mg/Tab Sanchal Salt84.952000 mg/Tab *Sodii biboras*11.345000 mg/Tab *Zingiber officinale Roscoe* 34.035000 mg/Tab	Tablet	Twice a day
2	*Berberis lyceum Royle*	AHMAREEN	QARSHI INDUSTRIES (PVT) LTD	Ammonium chloride 30.000000 mg/10 ml *Berberis aristata DC*. 200.000000 mg/10 ml *Cichorium intybus Linn*. 100.000000 mg/10 ml *Citrus limonum Risso* (Oil) 10.000000 mg/10 ml *Citrus limonum Risso* (Bark) 300.000000 mg/10 ml Ferrous ammonium sulphate 50.000000 mg/10 ml Jawahar mohra 10.000000 mg/10 ml *Nelumbium speciosum Willd.* 100.000000 mg/10 ml *Rosa damascena Miller* 100.000000 mg/10 ml *Santalum album Linn*. 26.660000 mg/10 ml *Strychnos nux*-*vomica Linn.* (Extract) 10.000000 mg/10 ml *Vitis vinifera Linn*. 200.000000 mg/10 ml Preservatives 0.000000 Q.S Saccharum Base 0.000000 Q.S	Syrup	Twice a day
3	*Achyranthes aspera L.*	HOOPINIL	QARSHI INDUSTRIES (PVT) LTD	*Achyranthes aspera Linn*.312.000000 mg/10 ml *Adhatoda vasica Nees*125.000000 mg/10 ml *Ephedra gerardiana Wall. ex* Stapf125.000000 mg/10 ml *Glycyrrhiza glabra Linn*.125.000000 mg/10 ml Khashkhash musaffa125.000000 mg/10 ml *Mentha piperita Linn*. (Extract) 2.500000 mg/10 ml *Pistacia integerrima J. L. Stewart ex* Brandis125.000000 mg/10 ml	Syrup	6 times a day
4	*Riccinus communis L.*	DAWA-E-MALISH	Hamdard laboratories Waqf Pakistan	*Celastrus paniculatus Willd.* (Oil) 0.500000 g/3 g *Cinnamomum* cassia Blume (Oil) 0.100000 g/3 g *Ricinus communis Linn*. (Oil) 1.500000 g/3 g Sea Salt0.320000 g/3 g Styrax benzoin Dryander 0.160000 g/3 g Wax0.320000 g/3 g	Liquid	3 g once daily
5	*Carthamus tinctorious* *L.*	NAMAK JALINOOS	Hamdard laboratories Waqf Pakistan	Ammonium chloride 15.500000 mg/500 mg, Black salt 15.500000 mg/500 mg *Carthamus tinctorius Linn*. 15.500000 mg/500 mg *Carum carvi Linn*. 15.500000 mg/500 mg *Cinnamomum cassia Blume* 15.500000 mg/500 mg *Cinnamomum malabathrum Batka* 15.500000 mg/500 mg *Cuminum cyminum Linn*. 15.500000 mg/500 mg *Cuscuta reflexa Roxb.* 15.500000 mg/500 mg *Foeniculum vulgare Miller* 15.500000 mg/500 mg Lake salt 30.750000 mg/500 mg *Piper nigrum Linn*. (White) 15.500000 mg/500 mg *Piper nigrum Linn*. (Black) 15.500000 mg/500 mg Rock Salt 185.000000 mg/500 mg *Valeriana officinalis Linn*. 15.500000 mg/500 mg *Zingiber officinale Roscoe* 15.500000 mg/500 mg	Tablet	2 tab once daily
6	*Solanum nigrum L.*	MADAMOL SYRUP	QARSHI INDUSTRIES (PVT) LTD	*Acacia arabica* (*Lam*.) Willd. 62.500000 mg/10 ml *Achillea millefolium Linn*. 62.500000 mg/10 ml *Adiantum capillus*-*veneris Linn* 62.500000 mg/10 ml *Cichorium endivia Linn*. 62.500000 mg/10 ml *Ficus bengalensis Linn*. 62.500000 mg/10 ml *Fumaria offcinalis* 62.500000 mg/10 ml *Gendarussa vulgaris* 62.500000 mg/10 ml Iron Compound 50.000000 mg/10 ml *Juniperus communis Linn*. var. saxatilis Pall. 62.500000 mg/10 ml *Melia azadarach Linn*. 62.500000 mg/10 ml *Nepeta ruderalis Ham.* 62.500000 mg/10 ml *Pimpinella anisum Linn.* 62.500000 mg/10 ml *Rubia cordifolia Linn.* 62.500000 mg/10 ml *Solanum nigrum Linn*. 62.500000 mg/10 ml	Syrup	2 teaspoon twice a day
7	*Aloe barbadensis Mill.*	BARRISAL	Hamdard laboratories Waqf Pakistan	*Aloe barbadensis Mill*. 1.000000 g/100 ml	Syrup	5 teaspoon thrice a day
8	*Ficus carica Linn.*	BERSEENA QURS	Hamdard laboratories Waqf Pakistan	*Acacia arabica* (*Lam*.) Willd. 23.520000 mg/Tab *Ficus caricaLinn*. 23.520000 mg/Tab *Melia azadarach Linn*. (Peel) 23.520000 mg/Tab *Melia azadirachta Linn* (Leaves) 23.520000 mg/Tab *Melia azadirachta Linn* (Bark) 23.520000 mg/Tab *Psoralea corylifolia* Linn. 23.520000 mg/Tab	Tablet	3 tab thrice a day
9	*Fumaria indica Linn*	ITRIFAL SHAHHATRA	Hamdard laboratories Waqf Pakistan	*Cassia angustifolia* Vahl. 0.075000 g/6 g *Emblica officinalis* Linn. (Dry) 0.150000 g/6 g *Fumaria indica* (Haussk) Pugsley (Leaves) 0.375000 g/6 g *Rosa damascene Miller* 0.045000 g/6 g *Terminalia chebula Retz*. 0.150000 g/6 g Sweetening agent and preservatives 0.000000 Q.S	Semi solid	6–12 g daily
10	*Datura stramonium L.*	HABB-E-MUMSIK TILAI	Hamdard laboratories Waqf Pakistan	Asphaltum 3.330000 g/Tab *Bombax ceiba Linn*. 16.660000 g/Tab *Centaurea behenLinn*. 8.330000 g/Tab *Corylus avellana Linn*. 2.660000 g/Tab *Datura stramonium Lin*n. 2.660000 g/Tab *Hyoscyamus niger Linn*. 2.660000 g/Tab *Juglans regia Linn*. 2.660000 g/Tab *Lactuca scariola Linn*. 2.660000 g/Tab *Lagenaria vulgaris* Ser 2.660000 g/Tab *Myristica fragrans* Houtt. (Seeds (nutmeg)) 1.330000 g/Tab *Myristica fragrans Houtt*. (Seeds (mace)) 1.330000 g/Tab *Papaver somniferum Linn*. 2.660000 g/Tab *Pinus gerardiana Wall ex Lam*. 2.660000 g/Tab *Pistacia vera Linn*. 2.660000 g/Tab *Prunus amygdalus Batsch*. 2.660000 g/Tab	Tablet	1–2 tab once daily

Worth and number of herbal industries is increasing day by day in Pakistan due to the trust of people on traditional medicines [56].

Ethno-medicines has a vital role in the industrialization [57]. Vivienne et al. stated commercial importance of medicinal plants in South Africa and reported 11 species of medicinal plants that are imported from India and other countries including Cinnamommum camphora. They also concluded that the size of the regional market of medicinal plants can be assessed by knowing the number of species traded. Commercial utilization of the medicinal plant is directly related to the degree of extinction [45].

## Future considerations

The review revealed that many of the important information like ethno preparation, habit and part used, of many important medicinal plants were not available in previous articles which can be due to the lack of interest of local youth to acquire the traditional knowledge from the ancestors and thus provision of incomplete information to the previous articles, further ethno-pharmacological research should be carried out to save the traditional knowledge and to take it to the light of science.

Medicinal plants in Pakistan contain great variety which can be used against a large number of skin ailments. Leaves, whole plant and roots are the most widely used parts in different ethno-medicinal preparations. Whole plant and root harvesting are the destructive type of techniques, it is important to protect the medicinal plants from exploitation. Although in Pakistan there is a strong traditional background supporting the use of these ethno-medicines against skin ailments but detailed ethno-pharmacological studies are not enough to support the folk claim. Majority of the studies have not documented other information regarding mode of preparation and dose of ethno-medicine. Therefore it is necessary to carry out a comprehensive study on ethno-pharmacology in Pakistan. It is important as the folk knowledge supplements a scientific investigations and penetrations with the primary information. Very limited number of studies provides toxic profile of these medicinal plants, toxicity studies should be carried out for these medicinal plants in animal system to establish a safe dose range.

More pharmacological studies (in vitro and in vivo) should be carried out on medicinal plants of Pakistan that are relatively unexplored or less explored. Mostly, the extracts were tested against different pathogens, for wound healing properties and for other skin conditions. However, very few classes of secondary metabolites and pure isolated components were tested. Therefore, it is imperative to conduct detailed phytochemical studies for the isolation of novel compounds.
